# Optical coherence tomography imaging biomarkers associated with neovascular age-related macular degeneration: a systematic review

**DOI:** 10.1038/s41433-022-02360-4

**Published:** 2022-12-16

**Authors:** Rachel L. W. Hanson, Archana Airody, Sobha Sivaprasad, Richard P. Gale

**Affiliations:** 1 Academic Unit of Ophthalmology, York and Scarborough Teaching Hospitals NHS Foundation Trust, York, UK; 2 Moorfields National Institute of Health Research, Biomedical Research Centre, London, UK; 3 Hull York Medical School, University of York, York, UK; 4 York Biomedical Research Institute, University of York, York, UK

**Keywords:** Predictive markers, Prognostic markers

## Abstract

The aim of this systematic literature review is twofold, (1) detail the impact of retinal biomarkers identifiable via optical coherence tomography (OCT) on disease progression and response to treatment in neovascular age-related macular degeneration (nAMD) and (2) establish which biomarkers are currently identifiable by artificial intelligence (AI) models and the utilisation of this technology. Following the PRISMA guidelines, PubMed was searched for peer-reviewed publications dated between January 2016 and January 2022. Population: Patients diagnosed with nAMD with OCT imaging. Settings: Comparable settings to NHS hospitals. Study designs: Randomised controlled trials, prospective/retrospective cohort studies and review articles. From 228 articles, 130 were full-text reviewed, 50 were removed for falling outside the scope of this review with 10 added from the author’s inventory, resulting in the inclusion of 90 articles. From 9 biomarkers identified; intraretinal fluid (IRF), subretinal fluid, pigment epithelial detachment, subretinal hyperreflective material (SHRM), retinal pigmental epithelial (RPE) atrophy, drusen, outer retinal tabulation (ORT), hyperreflective foci (HF) and retinal thickness, 5 are considered pertinent to nAMD disease progression; IRF, SHRM, drusen, ORT and HF. A number of these biomarkers can be classified using current AI models. Significant retinal biomarkers pertinent to disease activity and progression in nAMD are identifiable via OCT; IRF being the most important in terms of the significant impact on visual outcome. Incorporating AI into ophthalmology practice is a promising advancement towards automated and reproducible analyses of OCT data with the ability to diagnose disease and predict future disease conversion.

Systematic Review Registration: This review has been registered with PROSPERO (registration ID: CRD42021233200).

## Abstract (zh)

本综述有两个主要目的:1) 详细说明通过相干光断层扫描 (OCT) 可识别的视网膜生物标志物对新生血管性老年黄斑变性 (nAMD) 疾病进展和治疗反应的影响;2) 确定目前哪些生物标志物可由人工智能 (AI) 模型识别,以及该技术的应用情况.本文遵循PRISMA指南,在2016年1月至2022年1月期间使用PubMed数据库对同行评审出版物进行了系统检索.人群:经OCT成像诊断为nAMD的患者.设置:与NHS医院相似的环境.研究设计:随机对照试验、前瞻性/回顾性队列研究和综述文章. 在228篇文献中,对130篇进行了全文查阅,50篇因不属于本综述的范围而被删除,加入10篇来自作者清单的文章,最后纳入90篇.在确定的9个生物标志物中:视网膜内液 (IRF) 、视网膜下液、色素上皮脱落、视网膜下高反射物质 (SHRM) 、视网膜色素上皮 (RPE) 萎缩、玻璃膜疣、外层视网膜管状结构 (ORT) 、高反射灶 (HF) 和视网膜厚度中,有5个被认为与nAMD疾病进展有关:IRF、SHRM、黄斑、ORT和HF.部分生物标志物可以用目前的人工智能模型进行分类.与nAMD疾病活动和进展相关的重要视网膜生物标志物可通过OCT识别;IRF对视力结果的影响最为显著.将人工智能纳入眼科实践是OCT数据自动化和可重现分析的一个具有前景的进步,其拥有诊断和预测疾病转换的能力.

## Introduction

Age-related macular degeneration (AMD) is the most common cause of visual impairment in the developed world [1], with the late stage of the disease affecting an estimated 12.2% of individuals aged over 80 years in the UK [2]. Neovascular AMD (nAMD) is a severe form of AMD with an estimated 40,000 newly diagnosed cases yearly in the UK [2]. Although nAMD usually manifests initially unilaterally, the disease occurs in the unaffected fellow eye in 50% of patients within 3 years [3].

Following the pivotal ANCHOR, MARINA, CATT and VIEW 1/2 studies, gold-standard care for active nAMD involves treatment with regular intravitreal injection with an anti-vascular endothelial growth factor (anti-VEGF) drug, often for life [4–8]. Whilst such treatments do not cure nAMD they aim to prevent disease progression by halting leakage from abnormal blood vessels, which leak fluid and haemorrhage within and beneath the retinal layers. Anti-VEGF treatment successfully restores approximately half the initially lost visual acuity [9–12]. However, regular monitoring of disease activity to guide timely treatment delivery is essential for good treatment outcome.

Initial diagnosis and disease activity is imaged with optical coherence tomography (OCT), a tool for evaluating specific morphological retinal and sub retinal changes relevant for visual function and disease progression [13]. OCT is a non-invasive diagnostic method using infrared light in the 800–840 nm wavelength, providing real-time high-resolution imaging of the retina. Spectral domain-OCT (SD-OCT) acquires retinal images at rates up to 20,000 axial scans per second with almost 5 µm resolution, with some state-of-the-art machines even managing 40–70,000 scans per second [14].

OCT-guided treatment following an as required (*pro re nata or ‘prn’*) regimen was shown to be as beneficial as a fixed monthly regimen in the HARBOR study [15], However in real-world studies including AURA and LUMINOUS, *prn* outcomes fall short of this standard [16, 17] and proactive treatment with a variable treatment interval (‘Treat and Extend’) is now common place [18]. Although visual acuity is the primary outcome measure, whichever regimen is used, OCT-guided re-treatment decisions are the standard-of-care throughout the monitoring phase of care [5, 7]. This is because OCT markers of reactivation of MNV precedes visual changes, highlighting the importance of OCT biomarkers [19]. Furthermore, disease management is often lifelong with monitoring for disease activity in the first eye or for monitoring of development of the disease in the fellow eye. This results is significant burden for not only patients and care givers [20] but also health care systems [21, 22].

The ability to identify key OCT imaging biomarkers associated with nAMD may enable earlier diagnosis, better monitoring of the disease course, improved prediction of prognosis and even development of disease. Prompt treatment may also prevent the significant declines in visual acuity often seen in real-world studies [12, 19, 23, 24]. Identification of imaging biomarkers may also enable the treating physician to tailor personalised treatment to each patient’s individual disease need in order to provide adequate disease control, minimise recurrence and neurosensory damage and limit the number of invasive and costly interventions. Reliable biomarkers allowing prediction of disease progression may help to eventually reduce the substantial monitoring burden [13].

The key findings of the last review article outlined that fluid localisation offers superior prognostic value over central retinal thickness [13]. Specifically, intraretinal fluid (IRF) was deemed negative to visual performance whilst subretinal fluid (SRF) was associated with superior visual performance. Retinal pigment epithelial (RPE) detachment was also identified as a biomarker responsible for reduced visual performance and irresponsive to treatment with alterations to the neurosensory tissue associated with irreversible loss of function. The authors concluded that whilst a combination of these biomarkers may lead to personalised prognosis and management of the disease in the future, a fundamental problem remains that OCT produces a vast amount of information which cannot be meaningfully evaluated within a clinic.

Since the aforementioned comprehensive review of imaging biomarkers in nAMD [13] there have been major advancements in the utilisation of artificial intelligence (AI) technology to identify diseased from non-diseased retinal images [25]. The aim of this current systematic literature review is to detail the impact of retinal biomarkers identifiable using structural OCT imaging on nAMD disease progression, response to treatment and how the understanding of these biomarkers have evolved over the past 5 years. The impact of these retinal biomarkers will be assessed in terms of; (1) biomarker definition, (2) relevance to vision and other outcome measures, such as fibrosis or atrophy in the affected and second eye (and 3) relevance to disease control with treatment. Furthermore, the current systematic review will establish how many of these biomarkers can be identified from OCT images assessed by current AI models.

## Method of literature review

The methodology of this systematic review followed the Preferred Reporting Items for Systematic Reviews and Meta-Analyses (PRISMA) guidelines [26]. The protocol for this review is also registered with PROSPERO (registration number: CRD42021233200). As this is a review of published literature, there was no requirement to obtain ethical approval.

A biomarker refers to a quantifiable biological parameter that is measured and evaluated as an indicator of normal biological, pathogenic or pharmacologic response to a therapeutic intervention, as defined by the National Institutes of Health [27]. All biomarker definitions outlined in Table 1 are descriptions as seen on structural OCT imaging and may differ to those defined using other imaging modalities.

**Table 1:** Retinal biomarkers detectable with structural OCT, their description and a summary of their impact on nAMD.

Biomarker	OCT description	High risk for disease progression?	References	
Intraretinal Fluid (IRF)	Initial fluid manifests as diffuse thickening of the outer nuclear layer. With more severe fluid exudation, cystoid spaces may form appearing as round or oval hyporeflective areas. Larger cystoid spaces often contain tissue septae and may involve all layers of the retina [118].	Yes	Kelkar, 2021 Finn, 2021 Marquis, 2020 Chakravarthy, 2020 Ross, 2020 Hsia, 2021 Zhang, 2018 Ashraf, 2018 Kang, 2017 Pokroy, 2018 Kim, J.M. 2017 Abdelfattah, 2016 Waldstein, Wright et al., 2016 Lee, 2017 Jaffe, 2019 Alex, 2021 Waldstein, Simader et al., 2016 Lin, 2020 Lai, 2019 van Romunde, 2019	Ogasawara, 2018 Burchard, 2018 Kim, J.H. 2017 Casalino, 2016 Chatziralli, 2016 Waldstein, Philip et al., 2016 Segal, 2016 Klimscha, 2017 Tuerksever, 2021 Schmidt-Erfurth, 2020 Schmidt-Urfurth, Waldstein et al., 2018 Schlegl, 2018 Sappa, 2021 Von der Burchard, 2018 Keenan, Chakravarthy et al., 2021 Schmidt-Urfurth, 2020 Schmidt-Urfurth, Bogunovic et al., 2018 Riedl et al., 2022
Subretinal fluid (SRF)	Appears as hyporeflective spaces with pockets of fluid commonly accumulating between the neurosensory retina and the RPE [118].	No	Kelkar, 2021 Finn, 2021 Marquis, 2020 Chakravarthy, 2020 Ross, 2020 Zhang, 2018 Ashraf, 2018 Kim, J.M. 2017 Abdelfattah, 2016 Waldstein, Wright et al., 2016 Lee, 2017 Klimscha, 2017 Waldstein, Simader et al., 2016 Hsia, 2021 Lin, 2020 van Romunde, 2019 Ogasawara, 2018 Burchard, 2018 Kim, J.H. 2017	Chatziralli, 2016 Waldstein, Philip et al., 2016 Segal, 2016 Jaffe, 2019 Tuerksever, 2021 Schmidt-Erfurth, 2020 Schmidt-Urfurth, Waldstein et al., 2018 Schlegl, 2018 Aslam, 2018 Bogunovic, 2017 Sappa, 2021 Von der Burchard, 2018 Keenan, Chakravarthy et al., 2021 Schmidt-Urfurth, 2020 Schmidt-Urfurth, Bogunovic et al., 2018 Riedl et al., 2022
Pigment epithelial detachment (PED)	Appears as broad elevations of the RPE band relative to Bruch’s membrane [118] with three common subcategories. Serous vascularised PED (svPED) on SD-OCT are identified as hyperreflective structures underneath the RPE that represent the CNV and fill out only part of the PED cavity [56]. In avascular sPEDs, domed-shaped elevation of the RPE can typically be seen overlying a homogenously hyporeflective space, with Bruch’s membrane often visible as a thin hyperreflective line at the outer aspect of the PED. Vascularised sPEDs are thought to occur when growth of a CNV lesion in the sub-RPE space is associated with profuse exudation, creating a serous fluid compartment. They appear similar to avascular sPEDs on OCT however, in some cases small collections of solid material (the apparent fibrovascular proliferation) can be seen adherent to the outer surface of the RPE [118]. Fibrovascular PED (fPED) with underlying occult CNV on SD-OCT are identified as the PED lesion’s cavity appears to be completely filled out by the CNV membrane [56]. fPEDs may be accompanied by variable quantities of serous exudation and/or haemorrhage; as a result the slope of the PED may vary depending on its fluid content [118]. Haemorrhagic PED (hPED) appear as frank haemorrhage from proliferating blood vessels in the sub-RPE space may also result in the formation of a haemorrhage PED. As in sPEDs, elevation of the RPE is often dome-shaped although the slope of such elevations is often more acture in the context of profuse bleeding. hPEDs often occur as a result of tears in the RPE [118].	No	Kelkar, 2021 Finn, 2021 Marquis, 2020 Chakravarthy, 2020 Hsia, 2021 Zhang, 2018 Ashraf, 2018 Ferrara, 2017 Kim, J.M. 2017 Clemens, 2017 Chatziralli, 2016 Klimscha, 2017 Schmidt-Erfurth, 2020 Lai, 2019 Azar, 2018 Ogasawara, 2018 Burchard, 2018 Casalino, 2016 Abdelfattah, 2016 Cho, 2016 Waldstein, Wright et al., 2016 Lee, 2017 Waldstein, Simader et al., 2016 Fragiotta, 2017 Schmidt-Urfurth, Waldstein et al., 2018 Sappa, 2021 Keenan, Chakravarthy et al., 2021 Yim, 2020 Schmidt-Urfurth, 2020 Schmidt-Urfurth, Bogunovic et al., 2018	
Hyperreflective material (HRM)	HRM is described asing the sub-RPE space, therefore some groups use the term HRM rather than SHRM [43, 59]. Well-defined HRM is described as a region of high reflectivity in which boundaries are clearly delineated from the surrounding neural components of the retina whilst undefined HRM is described as a region of with low reflectivity with less well defined borders and therefore not easily distinguishable from surrounding neural components [43, 59]. Pigmentary-HRM is significantly associated with progression to advanced AMD, NV and GA [60], yet baseline SHRM has not been significantly associated with the development of macular/geographic atrophy [39].	Yes	Roberts, 2019 Kovacs, 2018 Ferrara, 2017 Kawashima, 2017 Pokroy, 2018 Casalino, 2016 Abdelfattah, 2016 Lee, 2017 Jaffe, 2019 Casalino, 2020 Alex, 2021 Aslam, 2018	
Drusen	Drusen are the hallmark of AMD, seen clinically as pale, yellow deposits. On OCT, drusen are seen as accumulations of material between the RPE and Bruch’s membrane [119]. However, morphological considerations such as shape, reflectivity and homogeneity should be considered when grading disease severity and risk of progression. Reticular Subretinal drusenoid deposits (RPD), also referred to as subretinal drusenoid deposits (SDD), are often seen on OCT as deposits located internal to the RPE, in contrast to traditional drusen which are located external to the RPE [119]. RPD can appear as diffuse accumulations of material on the RPE extending to the EZ (stage 1) or as shallow mound-like accumulations of material on the RPE elevating the EZ (stage 2). They can also form conical haystack-like projections from the RPE extending across the EZ (stage 3).	Yes	Lamin, 2020 Abdelfattah, 2016 Zhou, 2016 Fogar, 2016 Notomi, 2021 Guymer & Wu, 2020 Waldstein, Vogl et al., 2020 Nassisi 2018; 2019 Banerjee, 2020 Yim, 2020 Waldstein, 2020 Tsuji, 2020 Alquadah, 2020 Li, 2019 Schmidt-Urfurth, Bogunovic et al., 2018 Pfau, 2021	
Retinal pigment epithelial (RPE)	A consensus meeting classified atrophy into the following four categories [71]: Complete RPE and Outer Retinal Atrophy (cRORA): a region of hypertension of at least 250m in diameter; a zone of attenuation or disruption of the RPE of at least 250m in diameter; evidence of overlying photoreceptor degeneration; the absence of scrolled RPE or other signs of an RPE tear. Incomplete RPE and Outer Retinal Atrophy (iRORA): some hypertransmission is evident but is discontinuous; the RPE band is present but irregular or interrupted; interrupted ELM and EZ evidences photoreceptor degeneration; the INL and OPL exhibit subsidence.	No	Ebner, 2021 Oliveira, 2021 Pfau, 2020 Abdelfattah, 2016 Kim, 2019 Ferrara, 2017 Kim, 2016 Chen, 2021 Pfau, 2021	
Retinal pigment epithelial (RPE)	Complete Outer Retinal Atrophy (cORA): continuous nonvisibility of the EZ and interdigitation zone; severe thinning of the outer retina; in the setting of an intact RPE band; hypertransmission associated with RPE degeneration is intermittent. Incomplete Outer Retinal Atrophy (iORA): continuous ELM and detectable EZ disruotion in the setting of regression subretinal drusenoid deposits with detectable thinning of the outer retina, an intact RPE band and no hypertransmission.			
Outer Retinal Tubulation (ORT)	ORT is defined on OCT as hyporeflective, branching tubular structures with hyperreflective borders within the outer nuclear layer of the retina, often overlying fibrous scarring [120].	Yes	Kovacs, 2018 Zhang, 2018 Chatziralli, 2016 Jaffe, 2019 Schmidt-Erfurth, 2016	
Hyperreflective foci (HRF)	HRF are characterised as small, well-circumscribed hyperreflective dots in the neurosensory retina, adjacent to fluid lesions [86, 118]. Although HRF in the inner and outer retina and SRF have been associated with worse baseline VA, a recent study found no significant correlation between HRF at baseline and VA at 3-, 6- or 12-months [86].	Yes	Hsia, 2021 Chatziralli, 2016 Tuerksever, 2021 Waldstein, Vogl et al., 2020 Nassisi 2018; 2019 Fragiotta, 2017 Yim, 2020 Waldstein, 2020 Schmidt-Urfurth, Bogunovic et al., 2018	
Retinal Thickness (RT)	OCT machines have integrated software to automatically segment the retinal layers, from the inner retina (internal limiting membrane) to the outer retina (RPE or Bruch’s membrane). This has enabled quantification of changes in retinal thickness to be an easy process for clinicians to make swift judgements relating to disease activity. Thickness measures can be obtained at different locations with common terminology including central retinal thickness (CRT), foveal centre thickness (FCT) and subfoveal central thickness (SFCT).	No	Ebner, 2021 Lin, 2020 Kumar, 2019 Azar, 2018 Fan, 2018 Pokroy, 2018 Chatziralli, 2016 Waldstein, Wright et al., 2016 Jaffe, 2019 Weingessel, 2016 Tuerksever, 2021 Schmidt-Erfurth, 2016	Schmidt-Urfurth, Bogunovic et al., 2018 Schmidt-Urfurth, Waldstein et al., 2018

References identified in the systematic review evidencing their impact are also noted with those pertaining to biomarkers identifiable using AI listed in bold.

To be eligible for inclusion, studies had to (a) include patients with nAMD on anti-VEGF therapy, (b) assessment of OCT (biomarkers c) examine the association between these biomarkers and prognosis, and (f) language had to be English. Studies were excluded if (a) the study population was not defined as nAMD, (b) no description on influence of OCT biomarker during study monitoring. Records of research protocols, reviews, and abstracts from scientific meetings were excluded.

A comprehensive review of relevant peer-reviewed literature published between January 2016 and January 2022 was carried out using PubMed. The keywords for the search can be found in (Table 2). The authors also supplemented the search results with peer-reviewed published articles from their own inventory. These included grey literature such as unpublished data and conference proceedings.

**Table 2:** PubMed search criteria and the number of results found.

Search string	Search dates	Number of results
(wet age-related macular degeneration OR neovascular age-related macular degeneration) OR exudative age-related macular degeneration AND (optical coherence tomography OR colour fundus photography) AND (biomarker OR marker OR predictive factor OR diagnostic accuracy OR predictive model OR prognostic model OR prognostic markers)	01/01/2016 – 01/01/2022	228

### Study selection

The citations identified by the literature search were assessed for inclusion in two stages by two authors (RLH and RPG). In stage 1, RLH screened all titles and abstracts identified for inclusion in the review, selecting those pertinent to biomarkers in nAMD. RPG then conducted a secondary screening of the shortlisted articles to clarify the inclusion decision. In stage 2, the full-text of all shortlisted articles identified in stage 1 was assessed by RLH to ensure eligibility. RPG conducted a further secondary screening. Any disagreements between the two authors were resolved by discussion at each stage.

## Results

**Fig. 1:**
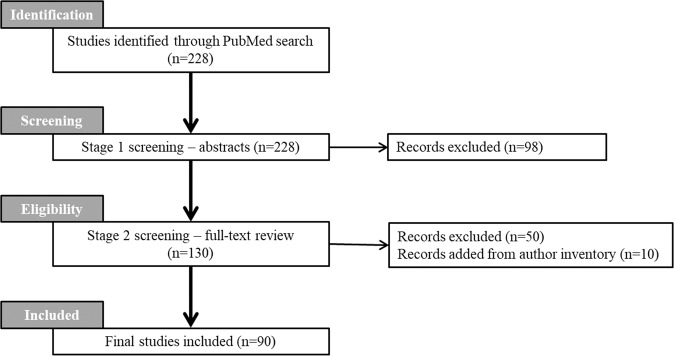
PRISMA flow diagram. Process of identification through to inclusion of the peer-reviewed articles in this systematic literature review.

In stage 1, titles and abstracts of 228 citations identified from the search were screened. Of these, 130 citations were screened in stage 2 via a full-text article review. Fifty articles were removed due to falling outside the scope of this review whilst 10 articles were added from the author’s own inventory including grey literature. This resulted in a total of 90 peer-reviewed articles included in this review (Fig. 1).

### Research synthesis

We evaluated the articles for the association of biomarkers for diagnosis or prognosis of nAMD by narrative synthesis utilising a best evidence synthesis approach. We did not design our review to present different levels of evidence. Meta-analysis was unable to be performed for any of the included studies due to high levels of methodological heterogeneity.

## Key retinal biomarkers

### Intraretinal fluid/intraretinal cyst

**Fig. 2:**
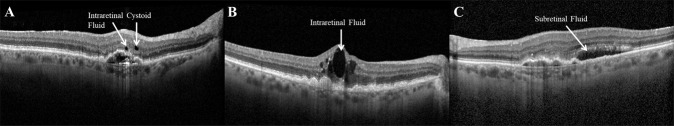
Optical coherence tomography images depicting the difference between retinal fluid in neovascular age-related macular degeneration. Intraretinal cystoid fluid (**A**) and Intraretinal fluid (**B**) appear as branching tubular structures that appear as round or ovoid hyporeflective spaces with hyperreflective borders in the outer nuclear layer, commonly overlying areas of PED or subretinal fibrosis [118]. Subretinal fluid (**C**) appears as hyporeflective spaces with pockets of fluid commonly accumulating between the neurosensory retina and the RPE [118].

The presence of intraretinal fluid/intraretinal cysts (IRF/IRC) (Fig. 2A, B) at baseline and during anti-VEGF treatment is repeatedly reported as significantly detrimental to visual outcome [28–38] although it has no significant association with developing macular/geographic atrophy (GA) [39]. Whilst quantification of IRC at baseline has been shown to predict 20% of the final best corrected visual acuity (BCVA) outcome at 12 months [40], worse BCVA at 1 year is also significantly associated with the presence of IRC at month-12 [29]. In patients with IRC at baseline, the BCVA improvement is less in those with persistent IRC at month-12 compared to those with resolved IRC [29] with some showing the resolution of IRC-related changes over time account for 40% of vision improvement from baseline to month-12 [40]. Comparing eyes with and without IRF, mean VA is significantly better when IRF is absent [34].

Location of IRC is also an important factor affecting visual outcome. Kang et al. reported that the presence of IRC in the inner nuclear layer (INLc) significantly predicted visual acuity (VA) changes from baseline to 24 months. Specifically, the presence of INLc and thinning of SFCT were associated with decreased BCVA at 24 months. Eyes without INLc (*n* = 35) showed improved logMAR BCVA from 0.550 (+− 0.273) to 0.368 (+− 0.274) (*p* = 0.045), however, eyes with INLc (*n* = 20) showed decreased BCVA from 0.708 (+− 0.347) to 0.971 (+− 0.523) (*p* < 0.001) over 24 months [41].

The effect of IRC on BCVA has also been shown to be dependent on the amount (mainly horizontal extension) and location (eccentricity from the fovea), with a significant correlation between BCVA and IRC volume (R2 = 0.44, *p* < 0.001) and IRC area (R2 = 0.57, *p* < 0.001) reported when IRC are centred on the fovea [40]. Evaluation of the CATT trial revealed that in 60% of patients with IRF at year 5, relative to the mean VA in eyes with no IRF (68 letters), mean VA was worse for eyes with extrafoveal IRF (57 letters, *p* < 0.001) and worse still for foveal IRF (44 letters, *p* < 0.001). The presence and foveal involvement of IRF is therefore independently associated with worse VA at year 5 [42].

Using baseline characteristics to predict visual outcome, the presence of IRF significantly accounts for the bulk of the predictive value [32]. Post-hoc analysis of data from the EXCITE study [37] also revealed IRC as one of three significant morphological features (along with subretinal fluid and posterior vitreous detachment) predictive of BCVA gain at month-12 (*p* = 0.05). Even following a simplified model analysis, the presence of IRC at baseline still significantly predicted BCVA change at month-12 (*p* = 0.03). The prognostic power of baseline characteristics has shown that good BCVA at month-12 is significantly associated with the absence of IRF [35].

Management of nAMD with anti-VEGF treatment is evaluated using the presence of retinal biomarkers such as IRF/IRC identified on OCT imaging. The prevalence of IRF/IRC was five-fold higher (24% v 5%) in injection compared to non-injection visits, with IRF/IRC present in 80% of injection clinics and absent from >85% of all non-injection, monitoring clinics [28].

The number of clinical visits during the maintenance phase (initial 4 months from diagnosis) has also been correlated with absence of fluid (both IRF and SRF) and gain in VA. Eyes with > _2 clinical visits noting the absence of IRF demonstrated significantly higher VA gains of five ETDRS letters compared with eyes with <2 clinic visits with absence of fluid with 2 ETDRS letters (*p* = 0.006). IRF without VA loss was only reported in 7.4% of injection visits [28].

A marked reduction in IRF is observed during the maintenance phase following commencement of anti-VEGF treatment [29, 43, 44]. Whilst some studies report the proportion of eyes with IRF remaining increased over 12 months from 18.2 to 30.6% [43], others have shown resolution of IRC at month 12 in eyes with IRC at baseline [29]. Another study assessed the outcomes of patients showing extended remission (ER), defined as the absence of haemorrhage, IRF, SRF on OCT and leakage on fluorescein angiography for 52 weeks after cessation of anti-VEGF. Results showed that of 830 eyes, 77 (9.2%) achieved ER during a median follow-up of 236 weeks. Importantly, the presence of isolated IRF at baseline predicted a shorter time to achieve ER (2.05-fold faster; *p* = 0.045) compared with eyes with combined IRF and SRF [45].

Although there are reports stating no significant difference between the anti-VEGF drugs ranibizumab and aflibercept, regarding the treatment response of IRC [29], one study has evaluated the effects of switching between the two [46]. The authors report that pre-switch structural changes on aflibercept negatively correlated with the post-switch response in morphological improvements to ranibizumab. The absolute values of IRF at the point of the switch predicted the degree of response to switching to ranibizumab; thus the more IRF present prior to the switch, the better the eye responded to the switch [46]. Yet, post-hoc analysis of the VIEW studies highlights that fluid resolution was consistently greater for aflibercept treated 4-weekly compared to aflibercept treated 8-weekly or ranibizumab. However, regardless of anti-VEGF drug, baseline IRC was associated with −2.77 letters from baseline at week 52 [38].

Persistent IRC at month 12 has been strongly associated with the treatment response of IRC at month 3. Taking both OCT morphology and treatment response into consideration gave the highest predictive power for persistence of IRCs at month 12. Authors could use baseline OCT morphology and the treatment response after loading injections to differentiate exudative IRC (due to active noevascular disease) from degenerative IRCs (architectural change leading to persistent fluid) and thereby better predict the persistence of IRCs at month 12 [29].

Baseline OCT factors have also been shown to predict response to bevacizumab treatment. Poor visual outcomes were associated with IRF (*p* = 0.020) and RPE loss (*p* = 0.009) when located in the subfoveal area. Following 3 injections of bevacizumab, IRF location and SRF width were the only biomarkers to explain 9.23% of the variation in the delta BCVA scores [47].

Post-hoc analysis of data from the EXCITE and VIEW studies revealed that IRC did not have any significant impact on differences in visual outcome as a function of treatment frequency at baseline. Patients performed better with frequent treatment than infrequent treatment by the same margin regardless of whether IRC was present (+4.6 letters) or absent (+4.3) at baseline [37, 38]. However, IRC has been shown to recur most rapidly between treatments, thus in eyes with a predisposition for IRC recurrence. Infrequent treatment may lead to a pronounced increase in IRC together with irreversible visual loss, and therefore such patients may benefit from more aggressive treatment [37]. Indeed, it has recently been shown that fluctuations in retinal thickness due to IRF have a negative impact of VA [48].

### Subretinal fluid (SRF)

Whilst some studies have reported a non-significant difference in visual improvement between eyes with or without SRF [34, 49], others report SRF (Fig. 2C) is in fact associated with superior baseline and outcome BCVA [32, 38, 47, 50, 51] and considered a significant predictor of BCVA gains at 12 months, but only when combined with the presence of posterior vitreous detachment [52, 53]. However, a recent report has shown a greater range of fluctuation of SRF during 12 months leads to lower BCVA at 12 months whilst a rapid improvement in SRF predicts better BCVA improvement at 12 months [44].

A study by Chatziralli et al. found that despite patients with SRF and no IRF exhibiting the numerically highest VA values throughout the study, final VA was not associated with the presence of isolated SRF; it is the presence of SRF and IRF [33] or SRF and PED [31] that are identified as specific prognostic factors for inferior visual outcome.

SRF is also attributed as a protective factor for the formation of GA compared with eyes without SRF at 24 months (8% v 33%) [34] whilst others have found no significant association between the presence of baseline SRF and macular/GA [39].

Evaluations of morphological features from the landmark CATT trial reveal that visual outcome also differs according to SRF location. At 5 years, relative to mean VA in eyes with extrafoveal SRF (57 letters), mean VA was better for eyes with foveal SRF (68 letters; *p* = 0.02) and similar to those without SRF (61 letters). When stratifying SRF thickness (0 µm, 1–25 µm and >26 µm), mean VA was also better for eyes with a foveal SRF thickness exceeding 0 µm compared with 0 µm (69 v 60 letters, respectively). However, increasing thickness of the subretinal tissue complex was also associated with increasingly worse mean VA [42].

Recently, Chakravarthy et al. have shown that eyes with at least 2 clinical visits with an absence of SRF demonstrated significantly higher VA gains compared with eyes with fewer clinic visits with the absence of fluid, with the prevalence of SRF six-fold higher (32% v 5%) at injection visits compared to non-injection visits [28].

Post-hoc analysis of data from the EXCITE study emphasised a significant interaction between SRF and treatment frequency (*p* < 0.001). Patients without SRF at baseline had higher BCVA gains with frequent monthly dosing (+12.3 letters) compared with infrequent quarterly dosing (+0.9 letters). However, when SRF was present at baseline, visual gains of patients receiving infrequent treatment were comparable to those receiving frequent treatment, with +2.6 letters in favour of infrequent treatment [37].

With regards to the type of anti-VEGF drug received by nAMD patients, a study by Segal et al. identified that in patients receiving bevacizumab, only SRF width demonstrated a significant positive correlation with BCVA (*p* = 0.018), suggesting as baseline SRF increases so does VA. Together, SRF width and SRF location explained 9.23% of the variation in BCVA scores [47]. Post-hoc analysis of the VIEW studies highlights that fluid resolution was consistently greater for aflibercept treated 4-weekly compared to aflibercept treated 8-weekly or ranibizumab. However, regardless of anti-VEGF drug, baseline retinal features influenced visual outcomes with patients with SRF exhibiting a gain of 2.11 letters at 12 months compared with patients showing IRC or PED [38].

Switching between anti-VEGF drugs is also required in some patients. A recent study has revealed a greater increase in SRF prior to switching from aflibercept to ranibizumab predicted a greater decrease thereafter. Also, the more fluid present prior to the switch, the better the eye responded to the switch [46].

Despite the benefits of anti-VEGF treatment for nAMD, discontinuation of treatment is sometimes requested by patients even with persistent or recurrent fluid. Evaluating the long-term visual prognosis in such cases revealed that eyes with only SRF showed significantly better VA at 24 months with a lower degree of visual deterioration during the follow-up compared with eyes with IRF with or without SRF (1.34 + − 0.38 v 1.79 + −0.60; *p* = 0.030) [51].

### Pigment epithelial detachment (PED)

**Fig. 3:**
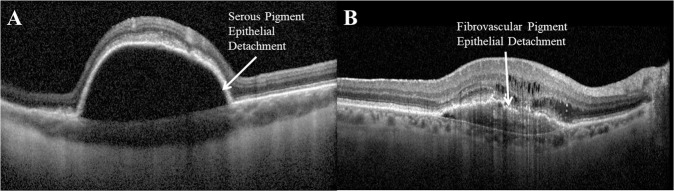
Two types of pigment epithelial detachment (PED) as seen on optical coherence tomography. Serous PED (**A**) appears as hyperreflective structures underneath the RPE that represent the choroidal neovascularisation (CNV) and fill out only part of the PED cavity [56]. Fibrovascular PED (**B**) are identified as the PED lesion’s cavity appears to be completely filled out by the CNV membrane [56]. fPEDs may be accompanied by variable quantities of serous exudation and/or haemorrhage; as a result the slope of the PED may vary depending on its fluid content [118].

Whilst the presence of PED at initial presentation has been associated with poor visual outcome [29, 33, 49, 54], some reports state no significant association with the risk of developing macular/GA [39] whilst others report specifically the PED width does predict disease progression [55]. Conversely, post-hoc analysis of the EXCITE and VIEW studies reveal that PED does not significantly predict BCVA gains at 12 months [37, 38]. Yet, the presence of PED *and* IRC at baseline was associated with less BCVA change from baseline to week 52 [38] whilst Ogasawara et al. conclude that PED *and* SRF are the specific prognostic factor for inferior visual outcome [31]. However, a distinct increase >5 ETDRS letters has been reported in patients with serous vascularised PED (svPED; Fig. 3A), identified on SD-OCT as hyperreflective structures underneath the RPE that represent the choroidal neovascularisation (CNV) and fill out only part of the PED cavity [56].

When comparing visual outcomes between patients with and without PED resolution after 12 months, Cho et al. report that despite no significant difference in the baseline BCVA, at 12 months BCVA was significantly better in patients with PED resolution [57]. Conversely Azar et al. reported that when a baseline PED > 250 µm is present at baseline, the initial response to 3 loading doses of ranibizumab is expected to be poor [54].

The prevalence of PED has been reported as 1.5-fold higher (58% v 36%) at injection visits compared to non-injection visits however, physicians appear to tolerate the presence of a PED in the absences of IRF or SRF without considering re-treatment [28].

Mean PED height has been shown to decrease significantly following 12-months anti-VEGF treatment [56, 57]. However, this effect seems to be driven by svPED height with no significant decrease in fibrovascular PED (fPED; Fig. 3B) height over the same time period [56]. Lower PED height at baseline has been associated with a greater probability of PED resolution after 12-months anti-VEGF treatment [57].

Considering the different anti-VEGF drugs available, Lai et al. outlined there is no significant difference between ranibizumab and aflibercept regarding the treatment response of PED [29] whilst Cho et al. report a greater probability of RPE flattening with aflibercept over ranibizumab treatment [57]. Nonetheless, when considering switching from aflibercept to ranibizumab, Marquis et al. report that a greater increase in PED prior to the switch predicted a greater decrease thereafter [46]. Patients who indeed switched to aflibercept showed a significant improvement in PED at 48 weeks [33]. Yet, post-hoc analysis of the VIEW studies highlights that fluid resolution was consistently greater for aflibercept treated 4-weekly compared to aflibercept treated 8-weekly or ranibizumab. However, regardless of anti-VEGF drug, baseline PED was associated with −1.8 letters from baseline at week 52 [38].

In contrast, a number of studies have reported that PED-related fluid is resistant to anti-VEGF treatment [34, 50] with a higher dosage adding no functional benefit in the landmark CATT, VIEW or HARBOR clinical trials [50]. Whilst a significant reduction in PED height is often observed following 12 weeks of treatment, this is not associated with significant improvements in VA. As such, treating PED to complete resolution may actually be detrimental to the patient [58].

### Subretinal hyperreflective material (SHRM)

**Fig. 4:**
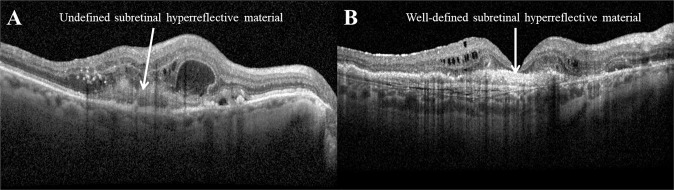
Two types of subretinal hyperreflective material (SHRM) as seen on optical coherence tomography. Undefined SHRM (**A**) is described as a region of with low reflectivity with less well defined borders and therefore not easily distinguishable from surrounding neural components [43, 59]. Well-defined SHRM (**B**) is described as a region of high reflectivity in which boundaries are clearly delineated from the surrounding neural components of the retina [43, 59].

The presence of SHRM at baseline is strongly associated with poorer BCVA compared with eyes without SHRM [30, 32, 42, 59]. A graduation of BCVA has been observed with the best VA outcome at month-12 in the absence of any HRM, the worst with undefined-HRM (Fig. 4A) and midway for well-defined HRM (Fig. 4B) [59]. The presence of SHRM and IRF also accounts for the bulk of the predictive value on visual outcome with the presence of SHRM at baseline the most significant predictor [32]. The weight of contribution of the baseline presence of SHRM increased with increasing follow-up.

Ferrara found that pigmentary-HRM (representing migration of the RPE), is significantly associated with progression to advanced AMD i.e., CNV or GA [60], yet baseline SHRM has not been significantly associated with the development of macular/GA by others [39].

Evaluation of 5-year data from the CATT trial reveals that although eyes with SHRM had worse mean VA, this was particularly the case if it involved the foveal centre (Foveal SHRM = 41 letters; extrafoveal SHRM = 63 letters; no SHRM = 72 letters). This was supported more recently by Alex et al. who reported worse VA when foveal SHRM exceeded 0.24 mm^2^ [30]. The CATT analysis also revealed that a greater loss of VA from year-2 to year-5 was also attributed to the incidence or worsening of 8 pathological features, including SHRM [42].

The prevalence of HRM has been shown to fall with anti-VEGF treatment [32, 43, 61] however, a study by Casalino et al. showed that over a 12-month period, whilst HRM decreased from 85.9% at baseline to 52.9%, along with evidence of undefined-HRM reduced from (53.7% v 7.4%) and SHRM (71.1% v 21.5%), there was an increase in prevalence of well-defined HRM (32.2% v 45.5%) and sub-RPE HRM, which was infrequent at baseline, increasing to 30.6% at month-12 [43].

A strong correlation between presence of fibrin on CFP and presence of HRM on OCT before initiation of anti-VEGF has been observed. This finding along with the marked reduction in the undefined component of HRM by month-1 suggests that the subset of HRM that is diffuse and located in the subretinal space is the result of an inflammatory reaction in early AMD. The observed increase of HRM with well-defined boundaries over time and that undefined HRM was frequently replaced by the well-defined variety after anti-VEGF supports the observation that anti-VEGF treatment induces a maturation of the neovascular complexes towards an organised tissue in which hyperreflectivity increases over time [59]. The finding that undefined HRM at month-12 had the poorest vision suggests a reactivation of the CNV complex and supports recommendations by Ores et al to consider undefined HRM as a qualitative criterion for retreatment [59].

Kawashima et al. propose that distinguishing between vascular (vSHRM) and avascular SHRM (aSHRM) could improve the ability of SHRM as a predictive factor for anti-VEGF efficacy. SHRM is composed of vascular components in 48% of cases and vSHRM is significantly associated with ELM disruption owing to SHRM in the outer retina and the presence of IRF at baseline. vSHRM is significantly associated with persistent SHRM after anti-VEGF which was also associated with a wet macular, suggesting that vSHRM could be a better predictive factor for less response to anti-VEGF than aSHRM [62].

A recent study by Roberts et al. used polarised-sensitive OCT (PS-OCT) to detect fibrous tissue within SHRM. The authors suggest that PS-OCT, a functional extension of SD-OCT, can “segment fibrosis as well as the RPE based on their birefringent and depolarising properties” [61]. Their results showed that PS-OCT can detect SHRM composition from polarisation preserving to birefringent material marking the angiofibrotic switch in 6 eyes. SHRM volume decreased significantly under anti-VEGF however, lesions unresponsive to therapy may progress to fibrosis as early as 3 months. Therefore, reduced SHRM thickness may be a prognostic marker for treatment response.

### Drusen

**Fig. 5:**
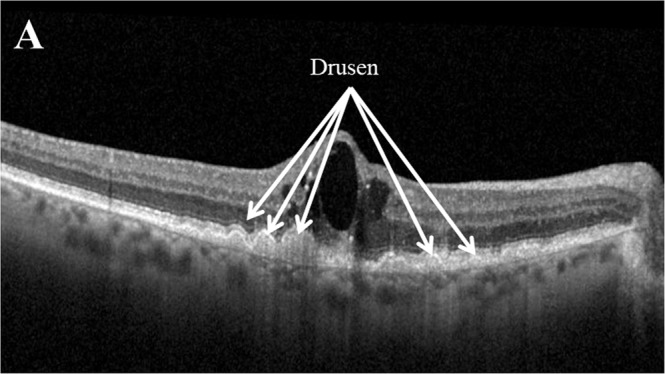
Example of drusen as seen on optical coherence tomography. Drusen of varying sizes, denoted by white arrows, appear on optical coherence tomography as accumulations of material between the RPE and Bruch’s membrane [119].

The presence of drusen (Fig. 5) has been associated with developing late AMD [63], with baseline drusen volume over 0.03 mm^2^ a significant predictor for developing late AMD in fellow eyes [39, 64], with a greater than 4-fold increase in risk at 1 and 2 years [63].

Increased drusen volume and drusen area have also been related to developing occult CNV [65] with baseline drusen volume significantly greater in eyes demonstrating CNV that in eyes without CNV [66]. Using the RPE-drusen complex to predict progression of intermediate AMD, Folgar et al. also revealed that each 0.1 mm3 increase in baseline drusen volume conferred 31% greater odds of CNV developing. Each 0.001 mm3 increase in baseline RPE-drusen complex abnormal thinning (RAT) volume conferred 32% greater odds of developing central GA whilst each 0.001 mm3 increase in RAT volume increased the odds of having GA after 2 years by 208% [66].

Waldstein et al. analysed OCT data from the fellow eyes of patients in the HARBOR study and found that in those eyes developing MNV, an increased mean drusen thickness of 29.6 µm was detected at the foveal centre. In eyes developing macular atrophy (MA), mean drusen thickness was 17.2 µm at the foveal centre. Eyes that did not develop advanced AMD within 24 months had an overall lower mean drusen thickness. Longitudinal modelling of drusen volume revealed eyes progressing to MNV featured a faster increase in drusen volume in the months before conversion compared with eyes developing MA and eyes not progressing. Drusen most frequently occurred in the foveal centre of eyes progressing to MNV whilst parafoveal drusen were seen in eyes developing MA. Drusen is therefore a significant biomarker of disease conversion to advanced AMD [67]. Similarly, fellow eyes of nAMD patients exhibiting soft drusen larger than 125 µm as well as the presence of medium drusen/pigmentary abnormality showed higher rates of nAMD occurrence within 5 years [68].

### Subretinal drusenoid deposits

Evaluating the association between subretinal drusenoid deposits and incidence of late AMD in fellow eyes of unilateral nAMD patients, the presence of baseline subretinal drusenoid deposits was significantly associated with a higher risk of developing nAMD [69]. Comparing dot, reticular and confluent subretinal drusenoid deposits, only dot subretinal drusenoid deposits was independently significantly associated with nAMD development whilst confluent subretinal drusenoid deposits was independently significantly associated with GA development [69].

### Retinal pigment epithelial (RPE) atrophy

**Fig. 6:**
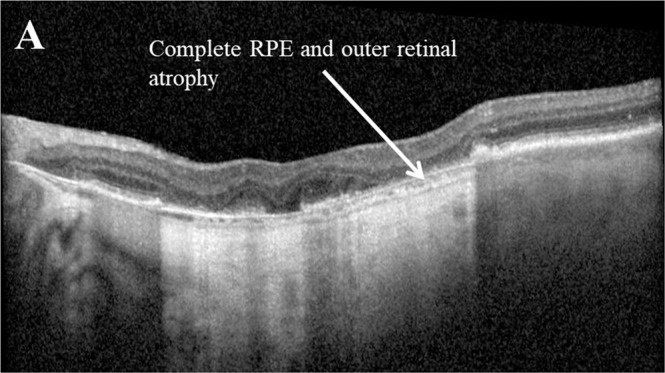
Example of complete retinal pigment epithelial and outer retinal atrophy (cRORA) as seen on optical coherence tomography. Complete retinal pigment epithelial (RPE) and outer retinal atrophy (cRORA) is defined on optical coherence tomography as a region of hypertension of at least 250 m in diameter; a zone of attenuation or disruption of the RPE of at least 250 m in diameter; evidence of overlying photoreceptor degeneration; the absence of scrolled RPE or other signs of an RPE tear (Sadda et al., 2018).

Retinal pigment epithelial (RPE) atrophy is characterised by a loss of retinal layers, RPE and choriocapillaris [70] with a new characterisation into four categories by a leading consensus group [71], descriptions of which can be found in Table 1. Of these new classifications, a recent study has shown that incomplete RPE and outer retinal atrophy (iRORA) has the highest incidence after 12 months, followed by complete outer retinal atrophy (cORA), incomplete outer retinal atrophy (iORA) and complete RPE and outer retinal atrophy (cRORA; Fig. 6) [72].

RPE atrophy is considered one of the primary factors in visual deterioration in type 3 neovascularisation. However, there are reports that focal RPE atrophy at the location of type 3 neovascularisation is in fact predictive of a favourable visual outcome (0.70 ± 0.48 LogMAR) compared with non-focal RPE atrophy (1.12 ± 0.68 LogMAR), with more participants loosing >3 lines ETDRS after 37 months [70]. Conversely, effects of RPE thickening are currently mixed with a suggestion this is significantly associated with progression to advanced AMD and neovascularisation [60] yet not associated with the risk of progression by others [73].

The localised future development of RPE atrophy has been significantly associated with follow-up time, distance to the atrophy boundary and eccentricity from the fovea and horizontal position [74]. The localised presence of treatment-naïve quiescent CNV is also associated with markedly reduced odds for the localised future progression of RPE atrophy [74].

Another early sign of RPE loss leading to subsequent RPE atrophy is RPE porosity [75]. Over a width of 20% of a polarisation-sensitive or SD-OCT b-scan, RPE porosity is classified as a series of RPE layer gaps with several RPE residuals between atrophic regions [75, 76]; a discontinuation of the RPE. A steady increase in RPE porosity and atrophy over time in patients with nAMD is suggested to substantiate chronic disease activity, possibly partly induced by intensive anti-VEGF treatment [75].

It has been suggested that frequent anti-VEGF injections are associated with the development or progression of RPE atrophy [39]. Over a 24 month period of anti-VEGF treatment, Kim et al. revealed that larger areas of RPE atrophy at month-4 and larger numbers of anti-VEGF injections were significantly associated with increased RPE atrophy [77] whilst higher numbers of anti-VEGF injections have also been associated with increased risk of developing cORA 12-months [72]. However, some have reported that reactivation of a type 3 neovascular lesion following the anti-VEGF loading phase is significantly lower with focal compared with non-focal RPE atrophy [70].

### Outer retinal tubulation (ORT)

**Fig. 7:**
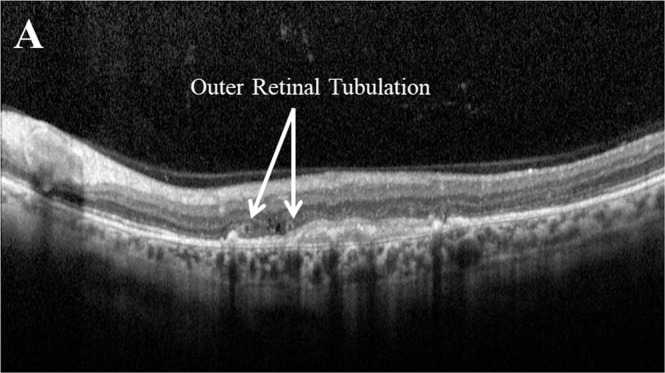
Example of outer retinal tubulation (ORT) as seen on optical coherence tomography. Outer retinal tubulations (ORT) are defined on optical coherence tomography as hyporeflective, branching tubular structures with hyperreflective borders within the outer nuclear layer of the retina, often overlying fibrous scarring [120].

The prevalence of ORT (Fig. 7) in nAMD increases over time and is associated with decreased VA [13]. Indeed, the five year evaluation of the landmark CATT study highlighted that eyes with ORT had worse mean VA compared to eyes without [42]. Conversely, others have reported that final VA is not associated with the presence of ORT [33].

Although the increasing prevalence of ORT over time has been suggested to occur irrespective of whether the anti-VEGF drug received is ranibizumab or aflibercept [78], there is evidence of a significant increase in ORT prevalence reported in patients who switched to aflibercept treatment [33]. Interestingly, considering other SD-OCT biomarkers, when SHRM was present at treatment initiation, the chance of developing ORT was 2.75 and 11.14 times higher in the ranibzumab and aflibercept groups respectively [78]. As such, a reduction in the number of anti-VEGF treatments is often expected upon the appearance of ORT [78].

### Hyperreflective foci (HRF)

**Fig. 8:**
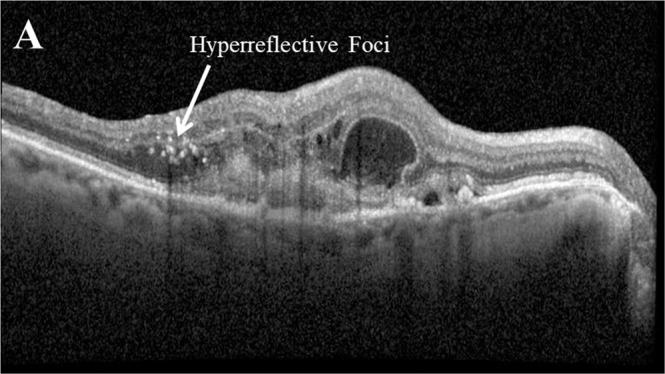
Example of hyperreflective foci (HRF) as seen on optical coherence tomography. Hyperreflective foci (HRF) appear on optical coherence tomography as small, well-circumscribed hyperreflective dots in the neurosensory retina adjacent to fluid lesions [86, 118].

There is current debate regarding the role of HRF (Fig. 8), including lipid exudation and inflammatory aggregates [79–81], migratory RPE cells that move toward the inner retina following disengagement from the RPE monolayer, whereas they transdifferentiate to express macrophage markers [82–84]. However, a number of studies have reported that HRF is significantly associated with the progression to late AMD [55, 64, 67, 85]. Longitudinal analysis of fellow eyes in the HARBOR study reported that a large mean HRF thickness was observed in eyes progressing to MA followed by eyes progressing to MNV with the mean volume of HRF within the central 3 mm foveal area fluctuating slightly during the months before conversion [67]. In eyes progressing to MNV, a slight steady increase of mean HRF volume was observed over time. Mean HRF thickness was larger when overlaying drusen, yet in eyes progressing to MA, the mean HRF thickness was also increased in areas unaffected by drusen. Similarly, two studies by Nassisi et al. also report a significant correlation between HRF area and progression to late AMD in eyes with intermediate AMD after 1 year [85] and that the presence of HRF was associated with a greater risk for progression to both atrophy and MNV [64].

The presence of HRF has also been significantly associated with the presence of other key biomarkers. The presence of IRF was significantly associated with the presence of HRF in the outer retina, inner retina and SRF layer; the presence of PED was significantly associated with HRF in the inner retina and the presence of SRF was significantly associated with HRF in the SRF layer [86]. Eyes with more HRF at baseline have shown significantly more reduction in CNV leakage area at month 12 with a rapid reduction in HRF at week 2 associated with lower CRT at month 12 [44]. HRF quantity is also reportedly significantly correlated with drusen volume at baseline and after 1 year [64, 85].

Following anti-VEGF treatment, a significant decrease in HRF in the inner retina, outer retina and SRF has been observed, with a faster decrease of HRF in the SRF layer [86]. A significant improvement over time in HRF has also been observed in patients switching to aflibercept treatment, with a prevalence of 38.7% (*n* = 173) at baseline decreasing to 32.4% (*n* = 145) at 48 weeks [33].

### Retinal thickness (RT)

**Fig. 9:**
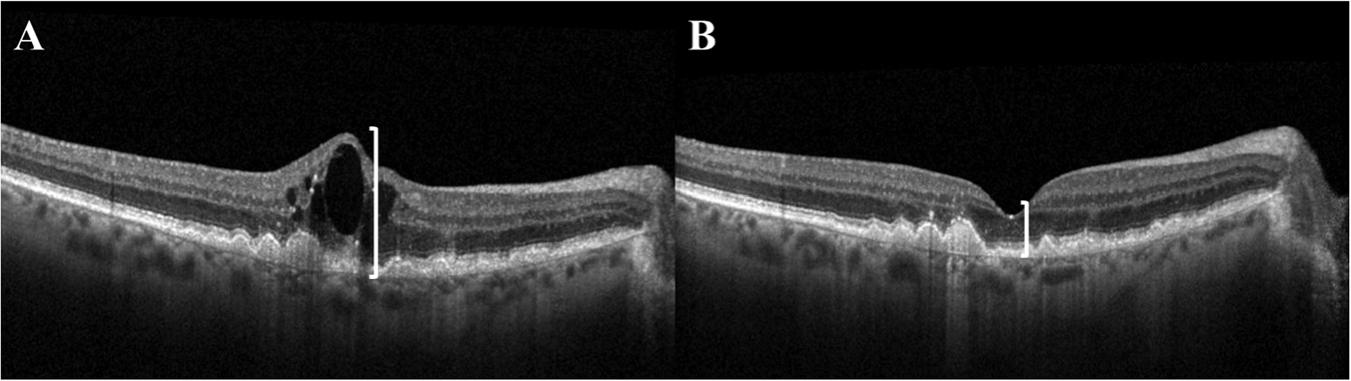
Example of retinal thickness (RT) as seen on optical coherence tomography. Cross-sectional optical coherence tomography image of the retina showing the difference in retinal thickness before (**A**) and after (**B**) treatment with anti-vascular endothelial growth factor in a patient with neovascular age-related macular degeneration. Vertical white lines denote the reduction in centre point thickness following treatment.

Considering that measuring RT includes changes occurring in different retinal layers, including SRF, IRF and PED, it is not surprising that reductions in RT are observed following anti-VEGF treatment [87] (Fig. 9) and that thicker SFCT is significantly associated with a greater number of injections [88].

Whilst earlier reports suggest that, particularly after the loading phase, CRT has poor sensitivity for detecting changes in BCVA compared with other retinal biomarkers [13, 53], Azar et al. reported that SFCT is a prognostic indicator of final BCVA [54] whilst an increase in CST has been significantly associated with poorer BCVA [33]. Analysis of the 5 year CATT data also highlighted that eyes with total retinal thickness >550 µm had significantly worse mean VA compared to eyes with <550 µm total retinal thickness (46 versus 61-65 ETDRS letters). This analysis also revealed that compared to a normal retinal thickness of 120–212 µm, eyes with very thin (<120 µm, 50 letters) or thick retinas (>212 µm, 54 letters) had worse mean VA, with 69, 50 and 54 ETDRS letters respectively [42]. Baseline central retinal volume and CRT have been shown to correlate significantly with BCVA at baseline, 3- and 12-months, with the correlation strengthening with increasing follow-up duration [32]. However, when large ranges in CRT fluctuation occurs during 12 months, BCVA is often lower at 12 months [44].

It has been suggested that a thinner SFCT is associated with baseline atrophy [88] with a general decrease of CRT, thinning of the choroid and RPE potentially signifying preliminary signs of atrophy development [75]. A thinner choroid has also be associated with eyes exhibiting macular atrophy (MA) at baseline and in eyes that developed new atrophy over 18 months, with a choroidal thickness ≤124 µm associated with a 4.3 times higher risk of MA [89].

## Artificial intelligence and OCT retinal biomarkers

The prevalence of AMD in the UK, including nAMD, is projected to double by the year 2050, affecting 1.23 million individuals [22]. Current ophthalmology services are facing unprecedented capacity challenges, with rising demand often outstripping the human and financial resources required to maintain high-quality and sustainable services. In light of the projected increase in demand from patients and capacity restrictions highlighted by COVID-19, new ways of efficiently and safely diagnosing and managing AMD is required to ensure timely treatment of patients, preventing unnecessary disease progression and ultimate vision loss.

Artificial intelligence (AI) uses artificial neural networks as a computational model to discover intricate structure and patterns in large, high-dimensional datasets such as medical images. A key feature of these convolutional neuronal networks is their ability to fine-tune on the basis of experience, allowing them to adapt to their inputs, thus becoming capable of evolving. This characteristic makes them powerful tools for pattern recognition, classification and prediction. For these reasons, incorporating AI technology within ophthalmology services may work towards alleviating some of this projected pressure on the NHS.

There is a growing amount of literature utilising AI in ophthalmology research, revealing sensitivity and specificity comparable to clinicians in identifying retinal disease from OCT retinal images [90–95]. Classification studies have revealed the ability of AI models to correctly classify retinal images depicting AMD from those depicting choroidal neovascularisation [96, 97], drusen [96, 97], diabetic macular oedema [96–99] and healthy retinas [100] with all models achieving high accuracies >90%.

Nevertheless, classifying AMD from OCT retinal images is not sufficient on its own in diagnosing and monitoring the disease. The ability to correctly identify retinal biomarkers indicating disease activity in AMD would truly aid clinicians in their monitoring process. The following section of this systematic review will discuss, of the key retinal biomarkers associated with AMD outlined previously, which are currently identifiable using AI technology.

### Biomarkers identified using AI

A number of studies have focused on the ability of AI to classify and quantify IRF, SRF and PED in nAMD amongst other retinal diseases. Utilising deep learning and convolutional neural networks (CNN) in the AI models, researchers trained an AI algorithm, referred to as the Vienna Fluid Monitor, to classify IRC, SRF and non-fluid regions for each pixel of an OCT image acquired from either Cirrus or Heidelberg OCT devices [101]. From 1200 images, 400 images were of nAMD, IRC and SRF were detected with high accuracy between the OCT devices with a mean area under the curve (AUC) of 0.92/0.98, mean precision of 0.73/0.94 and mean recall of 0.82/0.92 for IRC and SRF respectively. These accuracies were reported as falling within the range of the inter-observer agreement between certified retinal experts [101].

Similarly, concordance has been reported between retinal specialists and the Notal OCT Analyser (NOA) in two recent studies. Firstly, based on 155 OCT images from a tertiary referral retinal centre (Belfast Health and Social Care Trust, Belfast, UK), results highlighted an accuracy of 91%, sensitivity of 92% and specificity of 91% in identifying fluid denoting disease activity [102]. Secondly, based on 1127 scans from the Age-Related Eye Disease Study 2 10-year Follow-on Study (AREDS2-10Y), an overall greater performance was found for the NOA versus retinal specialists in identifying retinal fluid with an accuracy of 0.851/0.805, sensitivity of 0.822/0.468 and specificity of 0.865/0.970, respectively [103].

RetFluidNet is another AI model using an improved CNN-based architecture to segment three types of fluid abnormalities, SRF, IRF and PED. Trained and tested on SD-OCT images from 124 nAMD patients, a high accuracy of 95.53%, 80.05% and 92.74% was achieved in detecting SRF, IRF and PED respectively, revealing its ability as a fully automated method supporting early detection and follow-up [104].

AI has also been used to predict visual performance from the presence of IRF, SRF and PED. The most relevant biomarker for baseline BCVA was the horizontal extension of IRF in the foveal area, and IRF volume in the central 1 mm, whilst SRF and PED ranked low, irrespective of their macular location. The most relevant feature for predicting visual performance was found to be IRF area and volume and baseline BCVA [105]. Similarly, per unit of 100 nl, an increase in IRF was found to be associated with a mean reduction in BCVA of −4.08 ETDRS letters whereas SRF was associated with a superior BCVA of +1.99 ETDRS letters [50]. Conversely, a decline in VA is observed with increasing SRF and SHRM when the ELM intact. When the ELM is not intact, whilst VA is reduced, increasing SRF and SHRM reduces VA further albeit with a smaller gradient than with an intact ELM [106].

Recently, a team have proposed the use of three-dimensional volumes referred to as nanoliters (nL), to quantify retinal fluid via AI technology [50, 107]. A large data set of OCT scans from 4 separate studies (HARBOR: *n* = 24,362; Belfast: *n* = 4,673; Tel Aviv: *n* = 1,470; AREDS2: *n* = 511) were analysed using either the Vienna Fluid Monitor [101] or the Notal OCT Analyser [102, 103]. Both AI technologies provide rapid visualisation of the extent, location and severity of retinal fluid via heat maps along with displaying the estimated volumes of IRF and SRF in nL. The authors conclude this method offers a more precise measurement of disease activity, overcoming the qualitative descriptions normally used where fluid is typically graded as present/absent or severe/mild [107].

This research group have also used such automated quantified volumetrics of IRF and SRF to assess the impact of visual function in nAMD [108]. They report that a reduction of IRF load by 100 nL after the first treatment corresponds to a BCVA gain of 2 letters whilst a reduction in SRF load of the same amount corresponds to a BCVA gain of 6 letters. This suggests that IRF is associated with more permanent damage to the neurosensory tissue compared with SRF. Whilst the authors hypothesise that a higher potential for visual improvement may be seen from SRF resolution rather than IRF resolution, this mainly applies to foveal SRF which is less common in nAMD [108].

Likewise, it has been suggested that detection of IRF and SRF alone is not sufficient for disease detection, yet when combined, disease activity is detected with a sensitivity and specificity of 98.6% and 82%, respectively [109]. Further characterisation of IRF into exudative and degenerative cysts increased specificity to 100%. However, this group propose that changes in macular retinal volume (MRV) is a better detector with a sensitivity and specificity of 93.9% and 93.3%, respectively. Combined SRF and IRF detection correlates sufficiently with need for retreatment. Combining the detection of SRF with changes in macular retinal volume further improves diagnostic accuracy to a specificity = 93.3% and sensitivity = 93.9 without relying on IRF or IRF characterisation [109].

In a separate analysis using only OCT scans from the HARBOR study, this team used their AI technology to assess fluid resolution by treatment regimen. Results revealed that a low residual IRF was independent of a monthly or pro-re-nata (PRN) treatment regimen. Mean SRF volume in the monthly was less than the PRN. PED was resolved more intensively during a monthly than PRN treatment regimen [50].

Further analysis of a subset of the HABROR data by the same research group used AI to predict groups of low and high anti-VEGF treatment requirement in nAMD [110]. Classification of low- and high-treatment requirement subgroups demonstrated an AUC of 0.7 and 0.77, respectively. SRF volume in the central 3 mm was the most relevant feature for prediction with the highest predictive values at month 2. The authors conclude these results are a significant milestone for AI-guided management and predictions for treatment intervals for nAMD.

Custom deep-learning-based analysis pipelines have also been used to probabilistically forecast needed anti-VEGF treatment from SD-OCT. Using a random forest regression model, prediction of future anti-VEGF frequency was observed with an accuracy of 2.6 mean injections per year and 2.66 injections per year using an NGBoost model. RPE-drusen complex thickness in the central fovea was an important predictor across both models [111]. Likewise, SSG-NET, a sensitive structure guided network was used to predict short-term anti-VEGF requirements from 4944 OCT scans from nAMD patients. Verifying its clinical efficiency against two other deep-learning models and four ophthalmologists, SSG-Net achieved a greater performance overall with an accuracy AUC of 0.83, sensitivity of 0.692 and specificity of 1 [112].

### Predicting disease progression/conversion with AI

The potential for AI technology to predict progression to advanced AMD will be a significant advance in the monitoring process. To date there have been a number of studies assessing this in varying AI methods. A study by Schmidt-Erfurth et al. included 495 eyes of which 159 converted to advanced AMD, 114 to CNV and 45 to GA, within 2 years. Their machine learning model differentiated between converting and non-converting eyes with an AUC of 0.68 and 0.80 for CNV and GA respectively. The team identified that outer retinal thickness, HRF and drusen area were the most critical quantitative features of disease progression. Specific predictive hallmarks were mostly drusen-centric for progression to CNV while biomarkers associated with the neurosensory retina and age were predictive of progression to GA [113].

Banerjee et al. devised a hybrid sequential prediction model called Deep Sequence, as a platform to predict the risk of exudation within non-exudative AMD eyes over a short-term (3 months) and long-term (21 months) timeframe from longitudinal data [114]. The model was trained on 13,954 OCT images from the HARBOR study with a resulting high prediction performance AUC of 0.96 and 0.97 within 3 and 21 months, respectively. This model was then tested on a real-world set of 2854 scans from the Bascom Palmer Eye Institute. Results showed a high predictive performance at 3 months (AUC = 0.82) with a slight decrease in performance at 21 months (AUC = 0.68). Despite the drop in predictive performance over the long-term, the short-term performance could still have high clinical impact for disease monitoring.

The ability to predict conversion to nAMD in the second eye is another major advancement with AI. Yim et al. recently investigated this, training and testing their AI model on 5581 OCT scans from 2795 patients with unilateral nAMD. This novel AI model analysed the data in two stages; firstly, the model was trained on scans manually segmented into 13 relevant tissue types. A classification network was then applied to predict conversion to nAMD within the next 6 months. Secondly, the model was trained on the raw OCT scans allowing it to capture imaging features not yet segmented in stage 1. Results revealed a per-volumetric-scan sensitivity of 80% at 55% specificity and 34% sensitivity at 90% specificity. Higher conversion rates were seen in groups with greater drusen volume with the model substantially more sensitive when features known to be predictive are present, including HRF, drusen volume and fibrovascular PED. The authors conclude that their AI model can identify anatomical changes before conversion and high-risk subgroups [115].

Similarly, a different AI model also reported that drusen and HRF are biomarkers of disease progression. OCT data from 1,097 patients from the HARBOR study was used, focusing on patients with early or intermediate AMD in the fellow eye. During 24 months, 135 eyes developed MNV, 50 eyes developed MA and 333 eyes did not progress to advanced AMD. Mean drusen thickness was 29.6 µm/17.2 µm at the fovea and 25.8 µm/21.7 µm at 0.5 mm eccentricity in eyes progressing to MNV and MA respectively. At the same locations, mean HRF thickness was 0.072 µm/0.059 µm and 0.161 µm/0.227 µm for eyes progressing to MNV and MA respectively. The predictive value of HRF and drusen volume reveal for the development of MNV, the largest mean AUC was 0.66 obtained using drusen volume at 0.5–1.5 mm eccentricity with a similar mean at 0–0.5 mm eccentricity (AUC = 0.65). For the development of MA, HRF volume at 0.5–1.5 mm eccentricity had the largest mean AUC of 0.73 [67].

## Conclusion

This systematic review has highlighted a total of nine retinal biomarkers identifiable on structural OCT which have been regularly referred to as pertinent to nAMD in research articles spanning the last 5 years. Of these biomarkers, the most important, in terms of their significant impact on visual outcome is IRF. Moreover, fluctuations in IRF are detrimental to visual outcome and should be closely monitored when increasing the interval between anti-VEGF treatments.

Whilst qualitative assessment of SRF is generally associated with better visual outcome [116], quantitative volumetric analysis reveals that resolution of SRF following the first treatment is associated with greater visual acuity gains [108]. PED also appears not to affect final visual outcomes but is associated with an increased treatment frequency [52]. Whereas fPEDs and sPEDs appear to decrease in frequency over time, shallow, irregular elevation of the RPE does not change [43], drusenoid PED is significantly associated with progression to advanced AMD and NV [60]. Therefore, a residual PED without other accompanying signs of lesion activity is considered benign [28].

The presence of SHRM, HRF, baseline drusen and changes in drusen volume and drusen area are considered early predictors of disease progression and conversion to late AMD. SHRM has also been identified prior to the onset of overt features of neovascularisation on OCT [117] and as such, in eyes at high risk of nAMD, the presence of SHRM in particular should raise the suspicion of active choroidal neovascualrisation.

Based on this systematic review, it is clear that debate remains regarding the role of some biomarkers, including HRF. This is an important area to resolve not only to improve our understanding of this biomarker on the progression of nAMD disease but also to further our understanding the impact anti-VEGF treatment may have. There also appears to be a knowledge gap surrounding responses to anti-VEGF treatment and patients who switch anti-VEGF drugs. Interpreting why some patients are poor responders to treatment and the requirements needed for switching between treatments would better equip treating clinicians.

In conclusion, important retinal biomarkers pertinent to disease activity and progression in nAMD can be identified routinely from structural OCT imaging, providing an individualised management tool. However, emerging innovative imaging techniques such as OCT-angiography have the potential to uncover additional biomarkers not yet identifiable with OCT alone. Incorporating AI into ophthalmology practice is a promising advancement towards an automated and reproducible analysis of clinical OCT data. AI has the potential to review retinal images more efficiently, identifying regions of pathology in which clinicians can focus their attention. Research has shown that a number of the most pertinent biomarkers can accurately be classified using the current AI models not only to diagnose disease but also to predict future disease conversion. AI also provides promising improvements for grading retinal fluid which could lead to more tailored treatment on an individual level. This move towards measuring retinal changes quantitatively, in terms of volume or area, may ultimately produce a more accurate diagnostic tool but further clinical testing of this would be required.
